# The effectiveness of different patient referral systems to shorten waiting times for elective surgeries: systematic review

**DOI:** 10.1186/s12913-021-06140-w

**Published:** 2021-02-17

**Authors:** Dimuthu Rathnayake, Mike Clarke

**Affiliations:** Center for Public Health, School of Medicine Dentistry and Biomedical Sciences, Queen’s University Belfast, Institute of Clinical Science Block A, Royal Victoria Hospital, Grosvenor Road, Belfast, BT12 6BA UK

**Keywords:** Referral methods, Health systems, Elective surgery, Waiting time, Waiting list, Primary care practitioner, Systematic review

## Abstract

**Background:**

Long waiting times for elective surgery are common to many publicly funded health systems. Inefficiencies in referral systems in high-income countries are more pronounced than lower and middle-income countries. Primary care practitioners play a major role in determining which patients are referred to surgeon and might represent an opportunity to improve this situation. With conventional methods of referrals, surgery clinics are often overcrowded with non-surgical referrals and surgical patients experience longer waiting times as a consequence. Improving the quality of referral communications should lead to more timely access and better cost-effectiveness for elective surgical care. This review summarises the research evidence for effective interventions within the scope of primary-care referral methods in the surgical care pathway that might shorten waiting time for elective surgeries.

**Methods:**

We searched PubMed, EMBASE, SCOPUS, Web of Science and Cochrane Library databases in December-2019 to January-2020, for articles published after 2013. Eligibility criteria included major elective surgery lists of adult patients, excluding cancer related surgeries. Both randomised and non-randomised controlled studies were eligible. The quality of evidence was assessed using ROBINS-I, AMSTAR 2 and CASP, as appropriate to the study method used. The review presentation was limited to a narrative synthesis because of heterogeneity. The PROSPERO registration number is CRD42019158455.

**Results:**

The electronic search yielded 7543 records. Finally, nine articles were considered as eligible after deduplication and full article screening. The eligible research varied widely in design, scope, reported outcomes and overall quality, with one randomised trial, two quasi-experimental studies, two longitudinal follow up studies, three systematic reviews and one observational study. All the six original articles were based on referral methods in high-income countries. The included research showed that patient triage and prioritisation at the referral stage improved timely access and increased the number of consultations of surgical patients in clinics.

**Conclusions:**

The available studies included a variety of interventions and were of medium to high quality researches. Managing patient referrals with proper triaging and prioritisation using structured referral formats is likely to be effective in health systems to shorten the waiting times for elective surgeries, specifically in high-income countries.

**Supplementary Information:**

The online version contains supplementary material available at 10.1186/s12913-021-06140-w.

## Background

Awareness of growing waiting times in health care is not a recent phenomenon and the challenges of waiting times and waiting lists have been subject to a wide variety of health service research. Presentation with multiple comorbidities and complex clinical conditions are a direct result of the advancing biological age and increasing life expectancy of populations over recent decades [1, 2]. As a consequence, demand for surgery is growing and has exceeded the capacity of hospital services [2], leading to long waiting times and lengthy queues in many publicly funded health systems [3–6]. Lengthy waiting lists cause distress not only to the patients who are waiting but also to service providers. This has led to waiting times for elective surgeries becoming a major policy concern in many countries, especially those with health systems operated with public funds [7, 8].

Referral methods play a major role in providing appropriate care for patients in many health care systems. The quality and efficiency in referral systems in Lower and Middle Income Countries (LMIC) are often undermined due to system-related inefficiencies such as poor infrastructure, relevant materials/equipment and insufficient skilled human resource [9]. Despite having established superior services, the inefficiencies in referral methods of health systems in high–income countries are more pronounced [10] and the problem of long waiting times for elective surgeries more explicit in developed health systems. Delays in patients receiving specialised care due to inefficiencies in referral methods are purely a systems issue and could be resolved with streamlining the care pathways [11]. This could be considered as one of ‘Third delays’ according to the three delays framework because these patients have reached the hospital [12] nevertheless the first and second delays occurs prior to that when deciding and reaching the hospital. Unlike in private health service, the choice of a hospital and a medical specialist are limited in the public referral systems, because most public hospitals preferring to accept patients within their geographical catchment areas. Primary care practitioners are usually the gatekeepers who control access to specialised care in health systems where referral systems are restricted. In a typical surgical care pathway, primary care practitioners play a major role in determining which patients are referred to the particular surgeon or surgical clinic. The long waiting times being a common area of complaint for both patients and general practitioners, they sometimes have different perceptions of the value of the referral process [13]. Some countries practice fast-track referrals in certain elective surgical care pathways [14, 15], but the lack of clear specificity of the referral criteria has been repeatedly highlighted. Long waiting times for surgeries are also associated with higher risks of serious complications and death, especially among adults [16, 17]. Amongst the research that has been done to improve referrals, positive decision support systems incorporating clinical guidelines and the collaboration of specialists have been found to be effective for general practice [18]. The reassurance of the necessity of the referral to the patient is also important because the non-attendance of some patients at surgery appointments is a major factor in prolonging waiting times for them and for other patients [19].

Waiting times are a key performance indicator for many healthcare systems, used to encourage improved performance in healthcare institutions, with the aim of delivering high-quality care without unnecessary delay [7]. Many patients who wait a long time for their surgery are more likely to report problems, which have been associated with reduced quality of life [20]. Prolonged pain, discomfort, anxiety and disability are initial consequences for waiting patients. Alongside these impacts, patients in lower socioeconomic categories have reported worse outcomes in quality of life parameters when they are assigned to long surgical queues [21]. Economic evaluations have also found that the negative impact of patient waiting time on cost-effectiveness may be non-reversible [22].

Despite increased funding in recent years, the demand for many elective surgeries exerts a substantial and growing challenge [5]. Furthermore, even though many approaches have been attempted to shorten waiting times, these have not led to improvements or reductions in waiting times for elective surgeries [23]. The ability of hospitals to improve performance has often been restricted due to resource constraints, with the supply of surgery not accounting for the increase in demand. The COVID-19 pandemic has added considerably to this challenge, leading to pressure on healthcare institutions to move patients out of hospital [24]. The pandemic preparations in hospitals had drastically changed surgical priorities whereas certain elective surgeries have been postponed indefinitely [25].

Waiting lists are considered as a non-price rationing mechanism for coping with excess demand [26]. A large amount of literature is available on methods and strategies to reduce patient waiting times for elective surgeries, and health systems interventions are often multifactorial and multidimensional, making it difficult to measure their effectiveness. Recently, research focus has shifted to individual strategies. Various referral systems and policies were investigated in many studies that were targeted at reducing waiting times for elective surgeries, but rigorous evaluations in the form of systematic reviews are limited. A Cochrane Review published more than a decade ago analysed different approaches to improve referral systems to increase the efficiency and effectiveness of patient care [27]. A more recent systematic review of guidelines for elective referrals of adult patients to surgical specialists concluded that these improve the appropriateness of care [28]. However, a more up-to-date review is needed to help healthcare systems develop comprehensive protocols to establish effective and efficient referral systems in surgical care pathways [10]. To bridge the evidence gap in existing literature, this comprehensive systematic review will synthesise global evidence on policy strategies with a unique insight to effective primary care referral methods. The objective of this paper, therefore, is to review and summarise recent research evidence relevant to primary care referral methods as part of the surgical care pathways that aimed to shorten waiting time for elective surgeries of adult patients.

## Methods

This paper is based on one of the sub-reviews in a major systematic review which takes a holistic approach to summarise policies, strategies and interventions that might reduce long waiting times for elective surgeries. We have conducted and reported the review according to PRISMA statement [29]. The PRISMA flow diagram for the major review is attached as Additional file 1. The broad scope allowed the inclusion of many existing research papers, relevant to various aspects of the surgical care pathway and waiting times. The review was registered in PROSPERO (CRD42019158455). During the article selection process for the full review, we grouped different methods and strategies as they appear in eligible studies. For this paper, at the final stage of inclusion, we focused on studies pertaining to the management of referral systems with an intention to reduce long waiting times for elective surgery.

The electronic databases of PubMed, EMBASE, SCOPUS, Web of Science and Cochrane Library were selected for the search. Search terms were decided using MeSH headings and keywords for the scope of the full review. Major types of elective surgeries were searched for broader inclusion. After performing pilot searches, a detailed search list was finalised. The search strategy combined with three sets of search terms. The searches were run from 14th December 2019 to 7th January 2020 to include relevant articles published from January 2014 to December 2019 without language restriction. The search strategy used for PUBMED is presented in Additional file 2. We also checked the reference lists of included articles for additional relevant citations and there was no eligible articles. This EndNote citation management software was used for de-duplication.

Inclusion and exclusion criteria: All types of literature published as a full article were included if they reported eligible studies. This includes original research published in journals, reports, editorials and literature reviews from the healthcare sector, governments and other related sectors irrespective of the income level of the country of research. Where a study was based on outpatient departments, the referral intervention needed to be targeted at reducing the patient waiting time for elective surgery. Health system interventions are often tested in quasi-experimental studies, rather than randomised trials due to the complexity of the approaches being investigated and the diversity of outcome measurements. Therefore, we included a range of study designs, with a design-specific assessment of risk of bias [30]. Our main outcome variable of interest is waiting time and it includes both outpatient and inpatient time. It is defined as the time period between the date that primary care practitioner referred the patient to surgical clinic up to the date of the surgery is being performed. All quantitative and qualitative reporting associated with proxy variables of change (e.g. patient numbers, efficiency, and number of surgeries) were considered for data synthesis. Simulation and modelling studies were excluded because these might not provide a reliable guide to what would happen in real-world scenarios.

The surgeries require penetration of a body cavity are considered as major surgeries and all surgeries of abdomen, chest or cranium are major surgeries. Minor surgeries are generally superficial and do not require the penetration of a body cavities [31]. Eligible participants were adult patients (≥18 years) who had been referred to a surgical clinic for major elective surgery. Patients having emergency surgery or paediatric surgery were excluded, as were those awaiting cancer or cancer-related surgery. Although most eye surgeries are considered as minor surgeries, we included referrals for cataract surgery because this is one of the longest surgical lists commonly reported in many countries [32, 33].

Article selection and data extraction: As the first step, the title and the abstract of the retrieved citations were checked by one reviewer (DR) to select relevant articles. Articles that were potentially eligible based on their title or abstract were retrieved in full and assessed for eligibility and relevance. Each potentially eligible article was discussed with the second reviewer (MC) and an agreement was reached on inclusion or exclusion.

Quality assessment: The validity of the results of any systematic review of a health systems intervention depends on the methods used in the included studies (which may have used different designs), rather than universal experimental criteria [34]. Considering the variety of study designs identified for this review, we used assessment tools that were relevant to the included study design. The Cochrane ROBINS-I (Risk Of Bias In Non-randomised Studies - of Interventions) [35] provides a thorough assessment of risk of bias for non-randomized intervention studies. The AMSTAR 2 (A Measurement Tool to Assess systematic Reviews) is a critical appraisal tool for systematic reviews of randomised and non-randomised studies of healthcare interventions [36]. Finally, the CASP tool (Critical Appraisal of Skill Programme) was used to evaluate the observational studies [37]. Both review authors agreed on the quality rating for each included study.

## Results

**Fig. 1:**
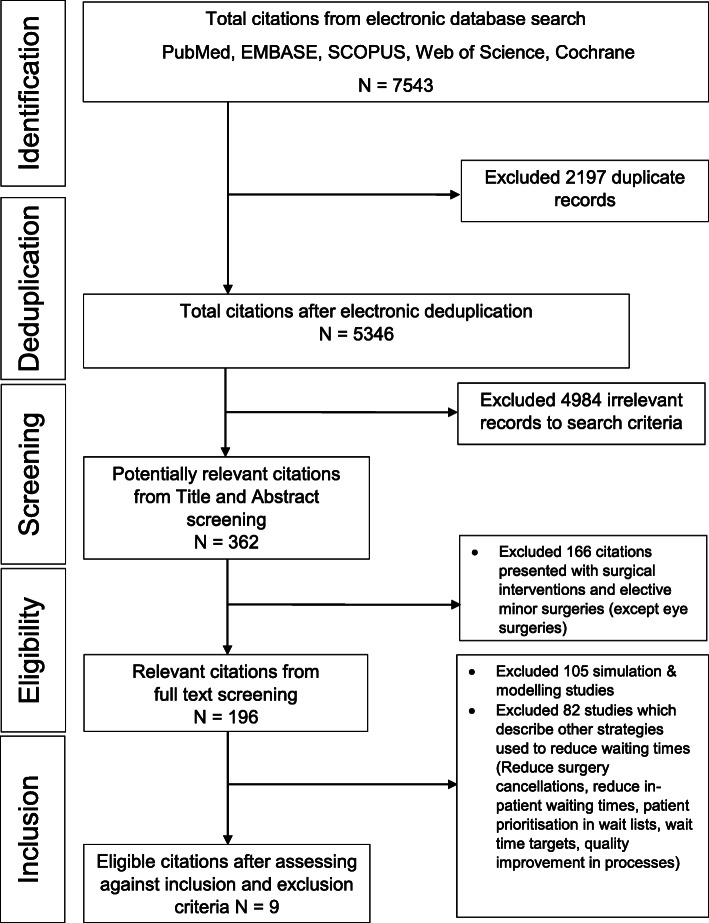
PRISMA flow diagram for the eligible article selection for the systematic review

The article screening process is shown in the PRISMA flow diagram (Fig. 1) and the PRISMA checklist is available as Additional file 3. The electronic search in the five bibliographic databases yielded 7543 records for the full review. This was reduced to 5346 after deduplication. During the title and abstract screening process, 362 citations were deemed potentially eligible for the full review. Of these, 196 relevant citations were obtained from full article screening, and, among these, 105 simulation and modelling studies were rejected. After grouping the citations into different strategies, three systematic reviews and six original studies (one randomised trial, two quasi-experimental studies, two longitudinal follow up studies and one observational study) were included for the final analysis of this sub-review, given their focus on interventions relevant to managing patient referrals as a way to reduce waiting time for elective surgery. The six original studies included were all based in high-income countries. There were three studies from Canada and one each from the USA, Israel and Australia. The summary of nine studies included is shown in Table 1.

**Table 1:** Summary of included studies

Characteristics	Number (*n* = 9)
**1. Publication year**	
2015	2 (22%)
2017	1(11%)
2018	3 (33%)
2019	3 (33%)
**2. Country of research**	
Canada	3 (50%)
USA	1 (17%)
Australia	1 (17%)
Israel	1 (17%)
*Three systematic reviews are excluded	
**4. Study design**	
Observational studies	3 (33%)
Systematic reviews	3 (33%)
Quasi-experimental studies	2 (22%)
Experimental studies (randomised trial)	1 (11%)
**5. Study setting**	
Institution	5 (80%)
Health system	1 (20%)
*The study setting was determined considering the research setting for the target intervention. The systematic reviews were excluded	
**6. Surgery types**	
Elective surgery	4 (45%)
Orthopaedic surgery	1 (11%)
Neurosurgery	1 (11%)
Eye surgery	2 (22%)
Bariatric surgery	1 (11%)

* indicate that how the studies were considered to categorise in the particular section

Summary of included studies: Nine studies were included in this review. The characteristics of the included studies are summarised below and further details are given in Tables 2 and 3. Of the three systematic reviews, one was a scoping review to describe strategies to reduce waiting times and the other two summarised existing evidence on increasing patient flow in elective care pathways.

**Table 2:** Study characteristics of the seven original research articles included for the review with the overall risk of bias evaluation

Author and year	Country	Research method	Elective surgery	Objectives	Research setting	Participants, Research period, and intervention	Conclusions & recommendations	Risk of Bias^a^
Damani et al., [38]	Canada	Quasi-experimental approach with pre-post cohort design	Total knee/hip replacement (TKR) surgery	To evaluate waiting time variations among surgeons, proportion of patients receiving surgery within benchmark, Influence across five dimensions of quality of care based on system- level and patient- centred outcomes (Quality accessibility, acceptability, appropriateness, effectiveness and safety).	Provincial health authority	Data were collected both before (June 2011–June 2012) and after implementation (September 3013–September 2014). Improve patient access to surgery by distributing referrals to the surgeon with shortest waiting time (next- available surgeon) and increase the proportion of patients treated within benchmark.	Intervention helped to improve accessibility by reducing waiting time variability among surgeons, all waiting times for TKR and increasing proportion of TKR within benchmark (5.9%).	ROBINS-I Moderate
Gabbay et al., [39]	Israel	Quasi experimental approach with historical prospective study	Cataract surgery	To evaluate the efficiency of referral triage system which schedules most cataract patients to surgery based on referral letters, with surgery done immediately following the preoperative examination.	A Tertiary referral hospital	Evaluated the performance of the new referral triage system (2015, 12 months) by studying the reason for day-of surgery cancelations against retrospective system.	The novel approach of preoperative triage using referral letters for scheduling surgery, thus minimizing both patient and physician time prior to surgery and direct referral could shorten both costs and time to surgery.	ROBINS-I Moderate
Coyle et al., [40]	Canada	prospective, blinded, randomized controlled study	Neurosurgery; Elective Lumbar Spinal Surgery	To evaluate whether a self-administered 3-item questionnaire (3IQ) could reprioritize referral appointments and reduce wait times.	Canadian academic tertiary care centre	280 patients included within 24 months. Randomly assigned to surgeon triaged and patient triaged two groups, assessed for re-prioritisation status and the waiting time.	Reduced the waiting time of intervention group and to identify non-surgical candidates for appropriate managements. Demonstrated the benefit of patient-reported assessments in prioritisation.	ROBINS-I Low
Do et al., [42]	Australia	Cross-sectional study with longitudinal follow-up	Cataract surgery	To determine the content and diagnostic accuracy of cataract referral letters and assessed whether referral information had sufficient detail to triage patients and inform surgical prioritization.	Two metropolitan public hospitals	A review of referral letters and hospital medical records was undertaken for a total of 400 (2014). Reviewed same after 1 year.	Current referral letters do not have sufficient detail to inform prioritization, and any efforts to prioritize waiting lists will require standardization of cataract referrals. Development of standard referral templates and resources to triage referrals may improve access to surgical services in a timely manner.	ROBINS-I Moderate
Loginov et al., [41]	USA	Observational study	Elective surgery	To examine patient perspectives on surgical case scheduling, referral and wait time.	Mayo Clinic	135 respondents completed the survey (2011–2016). The survey had three attributes; patient desired maximum waiting time, choice of date and option to change the surgeon.	There was a positive association between the maximum waiting times sufficient to discuss having another surgeon perform the procedure.	CASP Moderate
Diamant et al., [43]	Canada	Retrospective study	Bariatric surgery	To examine the impact of patient and operational factors on wait times in a multidisciplinary bariatric surgery program.	Toronto Western Hospital	1664 referred patients included for the survey (June 2008–July 2011). Waiting time associations screened for the 724 who underwent surgery.	Specific patient profiles and longer waiting are associated. Waiting time did not depend on BMI, age, sex and distance from the bariatric centre. Substance use was associated with longer preoperative evaluation. Certain types of patients should be identified early in the referral process.	ROBINS-I Moderate

^a^Risk of bias in intervention studies were assessed using ROBINS-I tool and observational study was assessed with relevant CASP checklists. Overall quality measurement was reported considering the all risk of bias domains for the particular research

**Table 3:** Study characteristics of the three systematic reviews included for the review

Author and year	Objectives of the systematic review	Search criteria			No of eligible articles and study designs^a^			Countries of included articles	Conclusions (%) & recommendations
		Data sources	Search date and search period	Languages	RI	NRI	OB		
Bachelet et al., [44]	Scoping review to identify and describe the interventions that have been implemented to reduce waiting times for major elective surgery and to synthesize the best available evidence on the effectiveness of some interventions that were prioritized by our ministerial counterpart	MEDLINE/PubMed, EMBASE, Cochrane Library, SciELO, DARE-HTA, and Google Scholar	All articles up to 2017 were searched in December 2017	Only English and Spanish	1	6	5	Canada, Costa Rica, UK, Spain, Nordic countries	All the studies had methodological limitations. According to the evidence found for this review, interventions most likely should be multidimensional, with prioritization strategies on the waiting lists to incorporate equity criteria, together with quality management improvements of the surgical pathways and the use of operating rooms, as well as improvements in the planning of the surgical schedule.
Damani et al., [45]	To review and summarise existing research evidence on the scope, use and implementation of SEMs for elective surgical services, specifically with respect to the influence of SEMs on patient flow and waiting times for elective procedures in adults and acceptability of SEMs to patients and providers (general practitioners (GPs) and surgeons).	MEDLINE, EMBASE, CINAHL, the Cochrane Database for Systematic Reviews, CENTRAL (Cochrane Central Registry of Controlled Trials) and Abstract Business Information (ABI)/Inform	All articles up to July 2016 was searched in June 2016	No restrictions	0	5	6	Canada, UK, Australia	This review demonstrates a potential ability for SEMs to improve timeliness and patient-centeredness of elective services; however, the small number of low quality studies available does not support firm conclusions about the effectiveness of SEMs to improve access.
Ballini et al., [46]	To assess the effectiveness of interventions aimed at reducing waiting times for elective care, both diagnostic and therapeutic.	Cochrane, MEDLINE, EMBASE, CINAHL, ABI Inform, the Canadian Research Index, The Science, Social Sciences and Humanities Citation Indexes, Pro quest, Trial Registries, Grey literature	All up to 2013	No restriction	3	5	0	Not reported	As only a handful of low-quality studies are presently available, it was unable to draw any firm conclusions about the effectiveness of the evaluated interventions in reducing waiting times. However, interventions involving the provision of more accessible services (open access or direct booking/referral) show some promise.

^a^*RI* Randomised Controlled Interventions, *NRI* Non Randomised Controlled Interventions, *OB* Observational studies

Starting with the original studies;

Damani et al. [38] reported a quasi-experimental study comparing a historical cohort (2397 patients recruited from 1 June 2011 to 1 June 2012) and a prospective cohort (2282 patients from 1 September 2013 to 1 September 2014) to assess the effects of a single-entry model (SEM) of referral to the next-available surgeon for total joint replacement surgeries in Canada. The results showed that the variability of waiting times among surgeons were reduced by 3.7 and 4.3 weeks for hip and knee replacements, respectively and there was a 5.9% increase in patients operated within the benchmark period.

Gabbay et al. [39] reported a quasi-experimental study with a historical and prospective study approach to evaluate the performance of a referral triage system through 2015 in Israel. They found that 44.4% of cancelled surgeries could have been prevented by a preoperative clinic visit and concluded that using a pre-operative triage system in referral letters for scheduling surgery could minimize both patient time and physician time prior to surgery.

Coyle et al. [40] reported a pragmatic, blinded, randomized trials with 227 consecutive eligible participants with an elective lumbar condition who were referred for consultation with a spine surgeon in Canada. Reprioritizing patients with a questionnaire reduced wait times for consultation appointments for patients who were eventually deemed to be surgical candidates. The odds of seeing a surgical candidate within the acceptable time frame of 3 months was 5.4 times greater for the intervention group. The authors concluded that it may be worth adding simple questionnaires to clinical care practices to better triage these patients on waiting lists.

Logvinov et al. [41] reported a survey of patients’ choices for the maximum waiting time sufficient to discuss having another surgeon perform the procedure in the USA. There were 135 respondents from 2011 to 2016. The results indicated that the average patient wanted to discuss the option of having another surgeon to perform their procedure would be 4 days or less.

Do et al. [42] reported a cross-sectional study with longitudinal follow-up conducted at two metropolitan public hospitals in Australia. A total of 400 sequential cataract referral letters were audited from August to September 2014 to benchmark against international prioritization tools. Results from the 12–15 months follow-up of these patients revealed that referrals for cataracts were poorly targeted, with almost half of all patients reviewed in the clinic not proceeding to surgery. The authors concluded that standardized referral templates may facilitate the improvement of referral pathways and shorten waiting times.

Diamant et al. [43] reported a retrospective study of the impact of patient and operational factors on waiting times for patients referred to a tertiary care centre in Canada for bariatric surgery between June 2008 and July 2011. The univariate and multivariate analyses showed that patients with active substance use and individuals who entered the program in more recent operational periods had longer wait times. They concluded that some patients could be identified at the time of referral as being at risk for longer wait times and that process-level decision-making for multistage bariatric surgical programs might affect timely access to treatment.

### Turning to the reviews

Bachelet et al. [44] reported a scoping review of studies of interventions that have been implemented to reduce waiting times for major elective surgeries. They searched six electronic databases up to December 2017 and included 12 eligible studies. They assessed the quality of the evidence with EPOC (The Cochrane Effective Practice and Organisation of Care) and GRADE (Grades of Recommendation, Assessment, Development, and Evaluation) tools, and rated all studies as low in overall quality. They concluded that there is a need for multidimensional interventions based on prioritization strategies, and quality management improvements of the surgical pathways and improvements in the planning of the surgical schedule.

Damani et al. [45] reported a review of the influence of SEM on waiting time for adult elective surgical services. They included 11 articles found from searches of six electronic databases up to July 2016. The authors used the Downs and Black checklist to assess the overall quality of the included studies. The results revealed a potential ability for SEMs to improve timeliness and patient-centeredness of elective surgical services.

Finally, Ballini et al. [46] reported a Cochrane Review of the effectiveness of interventions aimed at reducing waiting times for both diagnostic and therapeutic elective care. Eight studies were eligible and the overall quality of the evidence for all outcomes, assessed using the GRADE tool, ranged from low to very low. The authors assessed the risk of bias using EPOC criteria. They found that interventions involving the provision of more accessible services (open access or direct booking/referral) were likely to be effective.

Risk of bias in included studies: All included studies complied with the eligibility criteria for this review. The quality of evidence in the included articles was measured using the most appropriate of three tools: ROBINS-I Cochrane risk of bias tool (5 studies); AMSTAR 2, a critical appraisal tool for systematic reviews that include randomised or non-randomised studies of healthcare interventions, or both (3 systematic reviews) and CASP Critical appraisal skill programme for cohort studies (1 study).

**Fig. 2:**
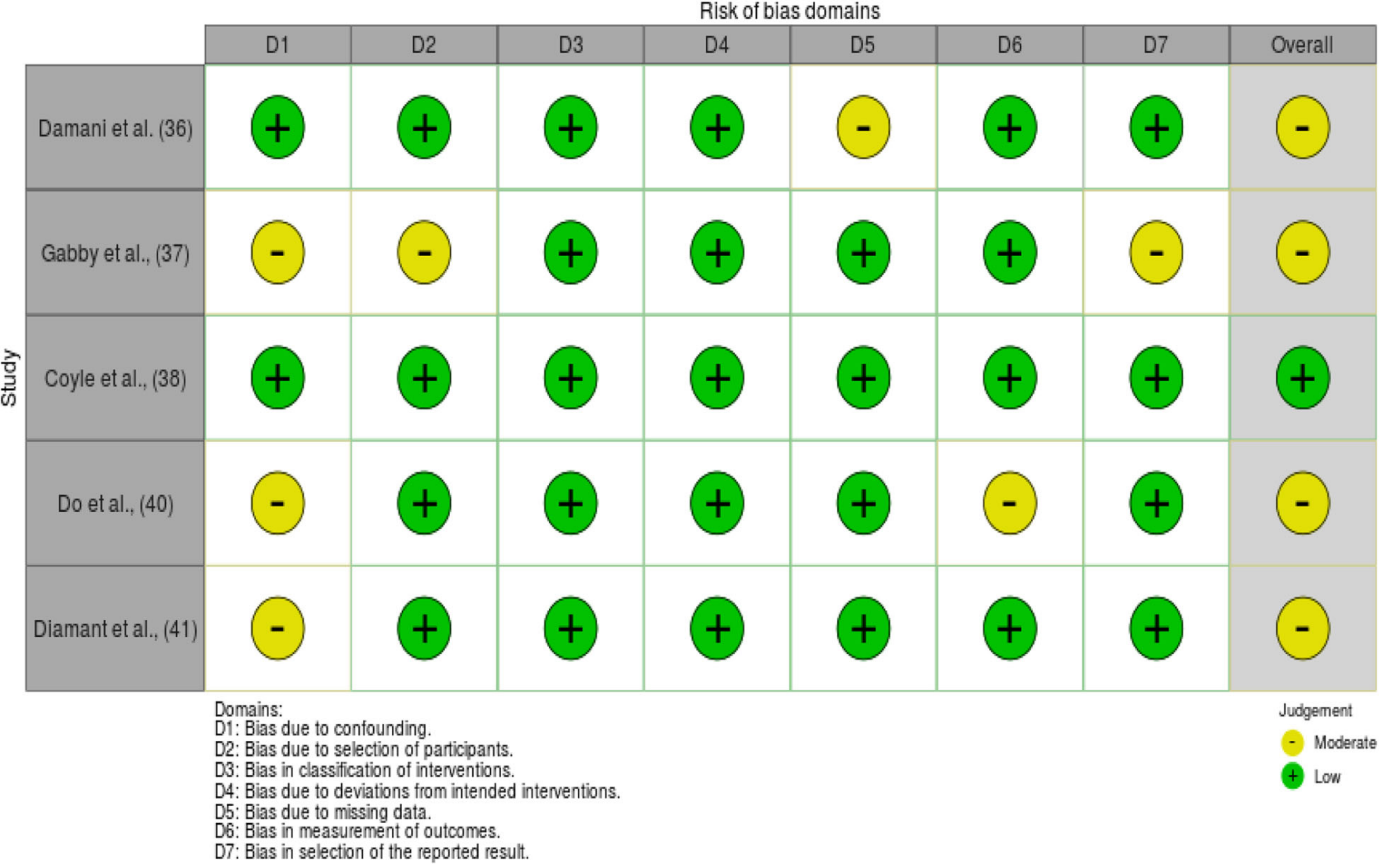
Traffic-light plot for the risk of bias domains in ROBINS-I for the five relevant studies

**Fig. 3:**
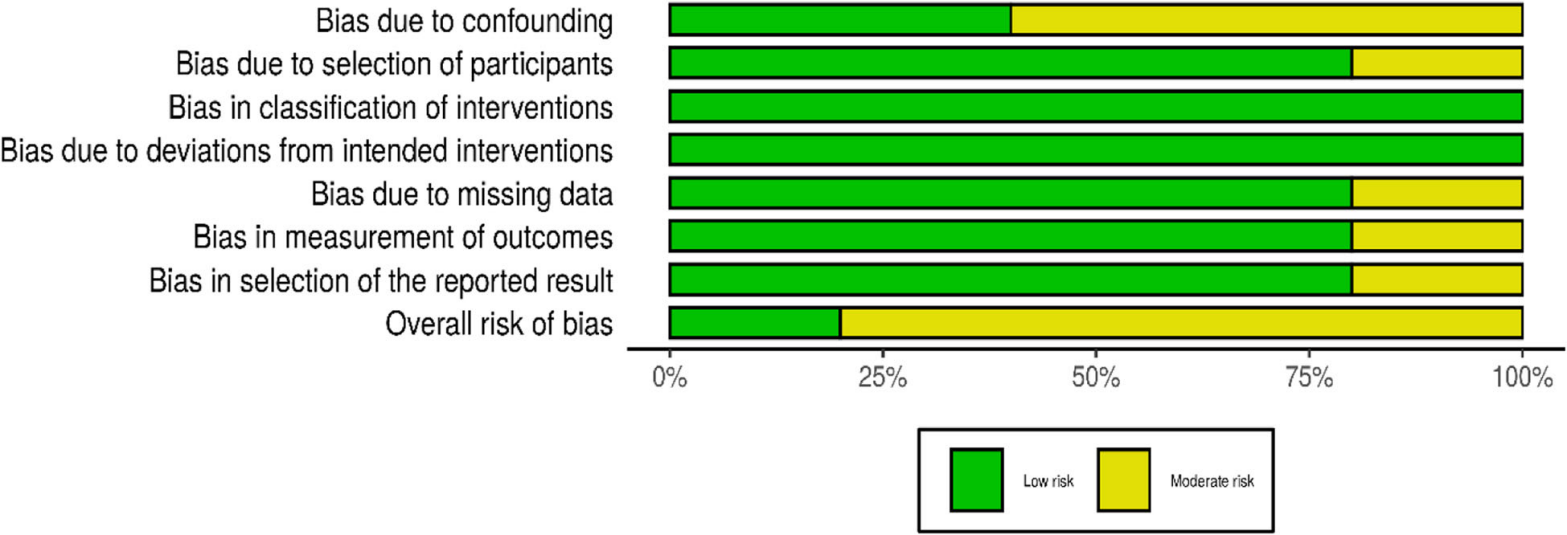
Bar diagram for each risk of bias domains in ROBINS-I for the five relevant studies

ROBINS-I: Of the five studies, a lower risk of bias was found for the randomised trial [40]. The other four studies were assessed as having the medium risk of bias overall [38, 39, 42, 43]. The details of the ROBINS-I evaluation are shown in Figs. 2 and 3.

One study used randomisation of participants [40]. The remaining studies had low to moderate bias due to confounding for baseline characteristics of the two groups [38, 39, 42, 43]. Since all patients in the selected cohort were included in all studies (without sampling), none of the studies had a significant bias in their selection of participants for the study. In most studies, patient data were extracted from regular administrative records, except for one study which was rated with a moderate risk of bias due to missing data [43]. Bias in the classification of the intervention was low in all studies, since the interventions were implemented as pre-test post-test design methods, where participants were not aware of the prioritisation scoring at the clinics. The outcome variable included in many studies is a time measurement associated with the waiting time, which was considered to be unbiased.

#### AMSTAR 2

One of the three included reviews was rated as high-quality overall [46] and the other two were rated as moderate [38, 44]. All review protocols had been registered and the reviews presented adequate searches for literature in relevant databases. All three reviews had assessed the risk of bias in their included studies and considered this when interpreting the results. Estimates of meta-analysis and assessment of publication bias was not applicable for any of the reviews. Our ratings for each domain for the AMSTAR 2 tool are shown in Table 4.

**Table 4:** AMSTAR 2 critical appraisal tool ratings for critical domains and overall confidence for risk of bias in included systematic review studies

Citation	Protocol registered	Adequacy of the literature search	Justification for excluding individual studies	Risk of bias from individual studies	Appropriateness of meta-analytical methods	Consideration of risk of bias when interpreting the results	Assessment of presence and likely impact of publication bias	Rating overall confidence
Bachlete et al. [44]	Partial Yes	Partial Yes	Yes	Partial Yes	NA	Yes	NA	Moderate
Ballini et al., [46]	Yes	Yes	Yes	Yes	NA	Yes	NA	High
Damani et al., [45]	Partial Yes	Yes	Yes	Yes	NA	Yes	NA	Moderate

#### CASP tool

A single study was assessed using the CASP checklist and the authors had reported important confounding factors for the study [41].

Synthesis of results: Meta-analysis was not applicable for this review, because of the heterogeneity in study designs and variability in how the outcome of interest was measured. Instead, we did a meta-synthesis with narrative analysis. The Table 5 summarises the relevant information on referral practices noted in the included studies.

**Table 5:** The information required during referrals to triage surgical patients as discussed in selected studies

Requirements of the referral information to triage patients	Selected studies					
	*Damani* et al.*,* [38]	*Gabbay* et al.*,* [39]	*Coyle* et al.*,* [40]	*Do* et al.*,* [42]	*Logvinov* et al.*,* [41]	*Diamant* et al.*,* [43]
1. Include relevant clinical details	√	√	√	√	√	√
2. Identify potential surgical candidate	√	√	√	√	√	√
3. Identify potential patients who need longer pre-op optimisation			√			√
4. Patients preferences for the scheduling time	√		√	√	√	
5. Patient reported health conditions			**√**			
6. Patient preference to change the surgeon	√				√	

## Discussion

Surgical referrals are considered as the interface between the referral provider (primary care practitioner) and the specialist (surgeon). The intended pathway for the patient to be referred to and what information needs to be recorded depends on the goal of the referral [47]. Certain referral systems have inherent barriers to efficient patient flow, meaning that better methods for patient triage and prioritisation at the referral stage might improve timely access and increase the number of consultations of relevant patients in surgical clinics. Having identified nice research studies published since January 2014, we discuss the relevance of their findings to this issue in this section.

Referral guidelines should be sufficiently standardised to allow those involved in making decisions about the referred patient to judge the appropriateness of the referral and to undertake a more detailed objective analysis of their needs. Referral letters that were unclear or not sufficiently informative led to dissatisfaction at surgery clinics [39, 42]. Tracing potential patients who required neurosurgical procedures with a simple questionnaire was effective at the referral stage [40]. It also improved timely access for surgical patients, while allowing non-surgical patients to be consulted at other clinics. There is uncertainty about whether the effects of these methods are similar for all specialties. For example, although preferential scheduling to prioritise low-risk patients for bariatric surgery was effective in fast-access for the surgery [43], it is uncertain if this approach would be effective for other conditions and types of surgery.

The proper reporting and adequate clinical assessment of the patient at their last consultation prior to surgery is important for informing the decision about whether to perform or cancel the elective surgery [48] and can prevent late cancellations of patients who have become unsuitable for surgery. Similarly, triaging patients for cataract surgeries at the referral stage with an informative referral note reduced cancellations on the day of the surgery [39]. The identification of patients who need longer pre-operative optimisation is important [43], to avoid allocating them a space that could be used for another surgery patient and to improve the efficiency of patient flow. There is supportive evidence to conclude that primary care physicians were at least as knowledgeable about most perioperative preparations for potential surgical patients as anaesthesiology residents, and this knowledge could be invested in achieving appropriate patient referrals [49].

Patient prioritisation for referrals is primarily based on clinical parameters. On the other hand, including non-clinical factors in the patients’ decision regarding a particular surgeon/hospital or surgery schedule with an open referral system was more effective [50] than the system deciding what is best for the patient. Adding patient preferences regarding a particular surgeon or surgery schedule to their referral notes were associated with improved access of patients to surgical care [41]. Similarly, the benefits of more accessible services for patients needing elective surgery through direct referrals have also been noted in the two systematic reviews [44, 46]. However, this comes with the challenge that patient preferences are variable due to many reasons. Identifying self-reported patient concerns were important and were associated with the willingness to undergo surgery for arthroplasty in elderly patients. The preferences of a patient towards a particular surgeon and selecting a hospital to admit is dependent on a myriad of factors and highly influenced by word-of-mouth recommendations [51]. The reputation of the surgeon, reputation of the hospital and experiences of peers are important factors and the ultimate decision is likely modified by the sociodemographic and cultural background of the patient to select the service provider [51].

Where there are multiple queues for surgical lists at the same surgery clinic, targeted patient referrals have had variable impacts on waiting times across different specialists. Single Entry Model (SEM) indicates the consolidation of multiple queues into a single queue. The application SEM while pooling referrals enabled patients to see the next-available surgeon for their procedure and improved timeliness and efficient patient flow [38]. The systematic review for SEM also showed a consistently positive impact on access-related variables for referral of patients to surgical clinics [45]. Recently, the combination of SEM and team-based care has been recommended as one way to confront the COVID-19 surgery crisis for efficient, fair, and ethical approaches in surgical care pathways [52]. However, although this review has found promising results in regard to balancing the variation of total waiting time with SEM, it can be difficult to implement SEM into a referral system due to inadequate stakeholder readiness and participation [53].

Streamlining the triage of patients during referrals with standardized referral templates and resources enhances timely access to surgical services [42]. Good cooperation between primary care practitioners and surgeons is important for a good referral system [54]. A substantial proportion of referrals could be dealt with through simple communication between the general practitioner and the consultant surgeon and, in some settings, general practitioners and specialists have worked together to produce guidelines for the types of patients that should be referred [28]. Not limiting to that, higher quality referral communications has instigated improved cost-effectiveness in surgical care [55].

Although the studies included in this review showed variable interventions with medium-high quality evidence, our findings have confirmed a consistently positive outcome with improvements to the timeliness of referrals at the primary care level in health systems of high-income countries.

## Limitations

This review is focused solely on the effects of referral methods that were intended to reduce waiting times for elective surgeries and which were reported in research studies published since 2013. It does not provide insights into the effects on waiting times of other forms of referral management that might be implemented for other purposes or on the research literature from before 2014. However, most literature suggests that the primary intent of referral management should be on prioritising care for patients most in need. Our limiting of the search to articles published in 2014–2019 may mean that we have failed to include some studies that would provide useful information. However, the use of systematic reviews published in this time window provides some insight into the older literature and our focus on recent evidence should increase the applicability of the results for contemporary practice in health systems. Our inclusion of observational studies helps to triangulate the constructs of interventional studies but brings with it concerns about their higher risk of bias. Finally, we have not been able to present meta-analyses of the effects of the interventions or determine if publication bias has affected our conclusions.

## Conclusions

On the basis of the available evidence, managing referrals by using triage and prioritisation of surgical patients is likely to reduce the waiting times for elective surgeries, by avoiding the overcrowding of surgical clinics with non-surgical referrals. Explicit and standard referral guidelines are more likely to be effective in selecting potential surgical patients if a structured referral formats are being used. In addition, using non-clinical information on a patient’s preferences for scheduling and switching their surgeon should also increase the timeliness of elective surgical care, and timeliness of patient flow may be increased with single entry models (SEM) for elective surgical services. Implementation of these interventions should mean that primary care practitioners and surgeons experience a more streamlined approach to elective care referrals.

In summary, this review has identified some interventions that could be implemented in the referral process for an adult elective surgery that might shorten waiting times and reduce the length of waiting lists specifically for high-income countries. However, more research is needed, ideally in the form of randomised trials to determine the effects of these interventions more precisely and to inform decisions around their cost-effectiveness. This may be especially important given the impact of COVID-19 on elective surgery waiting lists in many countries, and the depletion of resources for routine health care.

## Supplementary Information

**Additional file 1.**

**Additional file 2.**

**Additional file 3.**

## Data Availability

Additional data tables and figures are labelled attached.

## Abbreviations

PRISMA: Preferred Reporting Items for Systematic Reviews
ROBINS-I: Risk of Bias in Non-randomised Studies - of Interventions
AMSTAR 2: A MeaSurement Tool to Assess systematic Reviews
CASP: Critical Appraisal of Skill Programme
EPOC: The Cochrane Effective Practice and Organisation of Care
GRADE: Grades of Recommendation, Assessment, Development, and Evaluation
SEM: Single Entry Model
