# Supplementary material for: The effectiveness of different patient referral systems to shorten waiting times for elective surgeries: systematic review
Source: BMC Health Serv Res. 2021 Feb 17;21:155. doi: 10.1186/s12913-021-06140-w (PMC7887721; doi:10.1186/s12913-021-06140-w)
Supplement: Supplementary file 1 — Additional file 1. [file 12913_2021_6140_MOESM1_ESM.docx]

PRISMA flow diagram for the major systematic review and displaying sub-review headings

Total citations from electronic database search

PubMed, EMBASE, SCOPUS, Web of Science, Cochrane

N = 7543

Identification


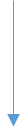

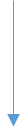


Excluded 2197 duplicate records

Deduplication


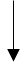


**Referral** **methods** **n = 9**

Resource Management n = 5

Outsource to private sector n = 4

Reduce surgery cancellations n = 37

Sub-reviews (*n denotes cumulative number)

Wait time target n = 3

Perioperative time management n = 20

Accurate and manipulate wait lists n = 3

Continuous process improvement n = 5

Patient prioritisation in wait list n = 17

Excluded 4984 irrelevant records

Excluded 166 irrelevant records

Potentially relevant citations from Title and Abstract screening N = 362

Total citations after electronic deduplication

N = 5346

Excluded 105 simulation/modelling studies

Eligible citations after assessing against inclusion and exclusion criteria N = 91

Relevant citations from

N = 196

Inclusion

Eligibility

Screening
