# Supplementary material for: The effectiveness of different patient referral systems to shorten waiting times for elective surgeries: systematic review
Source: BMC Health Serv Res. 2021 Feb 17;21:155. doi: 10.1186/s12913-021-06140-w (PMC7887721; doi:10.1186/s12913-021-06140-w)
Supplement: Supplementary file 2 — Additional file 2. [file 12913_2021_6140_MOESM2_ESM.docx]

**Search 1 – Date 14/12/2019**

**Total – 55**

**File – PubMed search 2.csv**

((((("waiting time"[All Fields] OR "waiting times"[All Fields]) OR "waiting lists"[All Fields]) OR "waiting list"[All Fields] AND ("2014/01/01"[PDAT] : "2019/12/31"[PDAT])) AND ("2014/01/01"[PDAT] : "2019/12/31"[PDAT])) AND ("2014/01/01"[PDAT] : "2019/12/31"[PDAT])) AND (("elective surgery"[All Fields] OR "elective surgeries"[All Fields]) AND ("2014/01/01"[PDAT]: "2019/12/31"[PDAT])) AND ("2014/01/01"[PDAT] : "2019/12/31"[PDAT])

**Search 2 – Date 14/12/2019**

**Total – 210**

**File – PubMed search 2.csv**

((((((("elective surgery"[All Fields] OR "elective surgeries"[All Fields]) OR "elective procedures"[All Fields]) OR "elective procedure"[All Fields]) OR "day surgery"[All Fields]) OR "day surgeries"[All Fields]) OR "ambulatory surgery"[All Fields]) OR "ambulatory surgeries"[All Fields]) AND ((((((((((((((((((((((("waiting list"[All Fields] OR "waiting lists"[All Fields]) OR "waiting time"[All Fields]) OR "wait times"[All Fields]) OR "patient wait"[All Fields]) OR "queue"[All Fields]) OR "appointment"[All Fields]) OR "schedule"[All Fields]) OR "prioritisation"[All Fields]) OR "prioritization"[All Fields]) OR "surgery list"[All Fields]) OR "theatre list"[All Fields]) OR (theater[All Fields] AND list[All Fields])) OR "schedules"[All Fields]) OR (theater[All Fields] AND ("time"[MeSH Terms] OR "time"[All Fields] OR "times"[All Fields]))) OR "theater time"[All Fields]) OR "appointments"[All Fields]) OR "theatre times"[All Fields]) OR "theatre time"[All Fields]) OR "patient register"[All Fields]) OR "patient registration"[All Fields]) OR "theatre allocation"[All Fields]) OR "queue"[All Fields]) OR "appointment"[All Fields]) AND ("2014/01/01"[PDAT] : "2019/12/31"[PDAT])

**Search 3 – Date 14/12/2019**

**Total – 2900**

**File – PubMed search 3.csv**

(((((((((((((((((((((((((((("waiting list"[All Fields] OR "waiting lists"[All Fields]) OR "wait times"[All Fields]) OR "waiting time"[All Fields]) OR "patient wait"[All Fields]) OR "queue"[All Fields]) OR "appointment"[All Fields]) OR "schedule"[All Fields]) OR "prioritisation"[All Fields]) OR "prioritization"[All Fields]) OR "surgery list"[All Fields]) OR "theatre list"[All Fields]) OR (theater[All Fields] AND list[All Fields])) OR "schedules"[All Fields]) OR (theater[All Fields] AND ("time"[MeSH Terms] OR "time"[All Fields] OR "times"[All Fields]))) OR "appointments"[All Fields]) OR "theatre times"[All Fields]) OR "theater time"[All Fields]) OR "patient register"[All Fields]) OR "theatre time"[All Fields]) OR "patient registration"[All Fields]) OR "theatre allocation"[All Fields]) OR "queue"[All Fields]) OR "appointment"[All Fields]) OR "cancel"[All Fields]) OR "cancellation"[All Fields]) OR "patient register"[All Fields]) OR "patient registration"[All Fields]) AND ("2014/01/01"[PDAT] : "2019/12/31"[PDAT])) AND ((((((((((((((((((((((((("abdominal surgeries"[All Fields] OR "breast surgeries"[All Fields]) OR "cardiovascular surgeries"[All Fields]) OR (("ear"[MeSH Terms] OR "ear"[All Fields]) AND ("nose"[MeSH Terms] OR "nose"[All Fields]) AND ("pharynx"[MeSH Terms] OR "pharynx"[All Fields] OR "throat"[All Fields]) AND ("surgical procedures, operative"[MeSH Terms] OR ("surgical"[All Fields] AND "procedures"[All Fields] AND "operative"[All Fields]) OR "operative surgical procedures"[All Fields] OR "surgeries"[All Fields]))) OR "endocrine surgeries"[All Fields]) OR "eye surgeries"[All Fields]) OR "general surgeries"[All Fields]) OR (geriatric[All Fields] AND ("surgical procedures, operative"[MeSH Terms] OR ("surgical"[All Fields] AND "procedures"[All Fields] AND "operative"[All Fields]) OR "operative surgical procedures"[All Fields] OR "surgeries"[All Fields]))) OR (("Head Neck"[Journal] OR ("head"[All Fields] AND "and"[All Fields] AND "neck"[All Fields]) OR "head and neck"[All Fields]) AND ("surgical procedures, operative"[MeSH Terms] OR ("surgical"[All Fields] AND "procedures"[All Fields] AND "operative"[All Fields]) OR "operative surgical procedures"[All Fields] OR "surgeries"[All Fields]))) OR "neurosurgeries"[All Fields]) OR "orthopedic surgeries"[All Fields]) OR "pelvis surgeries"[All Fields]) OR "reconstructive surgeries"[All Fields]) OR "thorax surgeries"[All Fields]) OR "transplant surgeries"[All Fields]) OR "urologic surgeries"[All Fields]) OR "oral surgeries"[All Fields]) OR "aesthetic surgeries"[All Fields]) OR "cataract surgeries"[All Fields]) OR "bariatric surgeries"[All Fields]) OR "orthognathic surgeries"[All Fields]) OR "knee surgeries"[All Fields]) OR "hip surgeries"[All Fields]) OR "plastic surgeries"[All Fields]) OR ((((((((((((((((((((((("abdominal surgery"[All Fields] OR "breast surgery"[All Fields]) OR "cardiovascular surgery"[All Fields]) OR "ear nose and throat surgery"[All Fields]) OR "endocrine surgery"[All Fields]) OR "eye surgery"[All Fields]) OR "general surgery"[All Fields]) OR "geriatric surgery"[All Fields]) OR "head and neck surgery"[All Fields]) OR "neurosurgery"[All Fields]) OR "orthopedic surgery"[All Fields]) OR "pelvis surgery"[All Fields]) OR "reconstructive surgery"[All Fields]) OR "thorax surgery"[All Fields]) OR "transplant surgery"[All Fields]) OR "urologic surgery"[All Fields]) OR "oral surgery"[All Fields]) OR "aesthetic surgery"[All Fields]) OR "cataract surgery"[All Fields]) OR "bariatric surgery"[All Fields]) OR "orthognathic surgery"[All Fields]) OR "knee surgery"[All Fields]) OR "hip surgery"[All Fields]) OR "plastic surgery"[All Fields])) AND ("2014/01/01"[PDAT] : "2019/12/31"[PDAT])) AND ("2014/01/01"[PDAT] : "2019/12/31"[PDAT])a
